# Reconstituted systems for studying the architecture and dynamics of actin networks

**DOI:** 10.1042/BCJ20253044

**Published:** 2025-05-23

**Authors:** Alice Cantat, Alexandra Colin

**Affiliations:** 1 Interdisciplinary Research Institute of Grenoble, Laboratoire de Physiologie Cellulaire & Végétale, CytoMorpho Lab, University of Grenoble-Alpes, CEA, CNRS, INRA, Grenoble, France

**Keywords:** actin, adaptability, confined environment, membrane interaction, microfabrication, micropatterns, reconstituted systems, size control, turnover

## Abstract

Actin, a ubiquitous protein essential for numerous cellular functions, is found in all eukaryotes. Despite extensive research across molecular to organismal scales, fundamental questions persist regarding the regulation of dynamic actin architectures, their interaction with membranes, and their mechanical properties. Characterizing the factors governing these processes presents significant challenges. This review emphasizes the value of simplified, reconstituted systems in addressing these unresolved questions. We particularly highlight the critical importance of macroscopic, network-level reconstitutions for tackling these issues. We first describe the available methodological toolkit for (1) controlling actin polymerization spatiotemporally and (2) confining actin networks within closed environments to examine boundary constraint effects or the impact of limited component availability on network properties. We then review studies employing these reconstituted systems to investigate how actin architecture influences various processes and how dynamic actin structures are established and maintained. Further, we discuss how network-level reconstitutions have enhanced our understanding of actin networks’ mechanical properties and their interaction with the lipid membranes. Throughout the review, we discuss future perspectives for each topic and explain how macroscale reconstitutions can provide deeper mechanistic insights into actin-related processes.

## Introduction

Actin is ubiquitous, being present in all sequenced eukaryotic organisms [1], and is found at very high concentrations ranging from 10 to 150 µM depending on cell type [2–5]. With the help of actin-binding proteins, actin filaments self-organize into different architectures for key functions in the cell [4,6,7]. Since its discovery in 1942 by Albert Szent-Györgyi [8], actin and its molecular partners have been intensively studied from the molecular scale to the organism scale [9–14]. Actin networks play key roles in shape maintenance, generation of forces for cell migration, and generation of forces to separate cells after division or endocytosis [10]. However, despite this intense research, many aspects of the actin cytoskeleton are still not fully understood, particularly at the mechanistic level. This includes the following questions:

- How are the sizes of actin structures defined?
- How is the balance between assembly and disassembly rates achieved to maintain the various sizes and turnover rates of the different actin networks?
- How are actin networks with various sizes and turnovers assembled from a common pool of monomers?
- How are those various network architectures dynamically transformed *in vivo* while keeping their integrity?
- How do actin filament mechanics or network geometry affect the activities of other actin-binding proteins?
- How does membrane geometry affect actin assembly? And reciprocally, how does actin architecture affect membrane properties?

Addressing these questions in a cellular context is extremely challenging. Indeed, the complexity of the cell’s interior, with hundreds of molecular actors present at the same time, makes it difficult to gain a mechanistic understanding of the processes at work in the cell. This is why, in this review, we will discuss the importance of using reconstituted systems to answer these major questions [15–18]. More specifically, we will explain why macroscopic reconstitutions, at the level of the networks, are key for addressing those questions. Here, we will focus exclusively on actin-based processes and how they have been investigated using purified proteins. Indeed, cell extracts provide an alternative approach for generating actin structures, as many relevant proteins are already present in the system: actin comet tails, contractile rings, and cortices have been successfully generated and studied in cell extracts [19–24]. However, studies using purified proteins offer distinct advantages for mechanistic investigations, as they allow for precise determination of the minimal set of components and their concentrations required for a given process.

## Importance of single-filament studies and CryoEM approaches to uncover molecular details of actin dynamics

Actin dynamics have been extensively studied *in vitro* through bulk experiments, which served to determine the effects of regulatory proteins on actin assembly and disassembly and to measure the rate constants of actin with its main partners [25–28]. The studies on individual filaments using first fluorescence microscopy and then TIRF (Total Internal Reflection Fluorescence) imaging [29,30] as well as the development of microfluidic systems [31] complemented these approaches. They allowed to study separately the steps of assembly [3,32–36], disassembly [37–42], and recycling [43–45], all key for actin dynamics. Experiments on individual filaments also identified specific properties such as branch formation [46,47], mechanical responses [48–50], or interaction with microtubules [51–54] that are not accessible with bulk experiments [55,56]. These studies have been complemented by single-molecule studies, which, for example, have given a better view of the branch formation by WASP and the Arp2/3 complex [57,58]. More recently, the drastic improvement of cryo-electron microscopy resolution [59] allowed to gain new molecular details about actin polymerization [60,61] and its dependence on actin-associated proteins [62–66]. Altogether, these studies have provided valuable insights into the interactions between actin and its binding proteins, the kinetics of their associated reactions, and other molecular mechanisms involved in these interactions. However, to understand how these proteins work together to control the dynamics of actin networks—and to address the key unresolved questions mentioned earlier—it is essential to extend reconstitution studies to the mesoscale. This scale bridges the gap between discrete molecular complexes and the intracellular environment [67–69].

## Why go for macroscopic reconstitutions?

Reconstituting dynamic actin networks in vitro at the network level offers several advantages. First, it is well-established that actin-binding proteins can exhibit different behaviors depending on the architecture of the network they interact with [6]. Therefore, reconstituting networks is essential for understanding how these proteins bind differently depending on the network’s structure. Additionally, reconstituting actin structures in three dimensions has revealed notably how applied forces influence network architecture and biochemical dynamics [70,71]. It is also widely recognized that physicochemical parameters play key roles in shaping the properties of the cytoskeleton within the cell [72–76]. Factors such as macromolecular crowding and viscosity [68,69,73,77] or energy availability [69] are, for example, critical parameters to consider when studying cytoskeleton-based processes. Finally, the effect of local and global depletion of actin and actin-binding proteins on the size and dynamics of actin networks is also an important factor to consider.

### Importance of the environment: the cell as a “constrained” environment

In cells, the reaction space is in the micrometer range. This implies that the number of molecules is small and potentially limited and that the boundaries can be reached by the various structures built into the cell. To assess the impact of these physicochemical parameters on cellular processes, the reconstitution of an environment mimicking the cell’s cytoplasm is of paramount importance. This environment is often established with confinement, achieved through microwells, water-in-oil droplets, or vesicles [78]. Most studies employ confinement for mechanical purposes, for example, to observe how it affects actin filament curvature [79–83]. In this case, the boundary condition is introduced to guide the spatial organization of the system. Recently, some studies have proposed using confinement to limit the amount of available components (similar to cellular conditions) and to examine how this global limitation affects the maintenance of actin dynamics over extended time periods and the coexistence of competitive actin networks [84,85]. The confinement of actin structures will also be critically important for studying the effects of local protein depletion mentioned previously.

## Toolbox: what kind of macroscopic reconstitutions?

To properly reconstitute actin networks, actin polymerization must be spatiotemporally controlled. In cells, actin polymerization and consequently the formation of the actin network is regulated locally by various activators and nucleators [86]. Over the years, different laboratories have developed various methods to spatiotemporally control the actin polymerization. Currently, the main tools used in reconstituted systems include beads and micropatterns, covered with lipids and/or activators for actin polymerization (WASP or formins, for example).

### Protein photoactivation

In order to localize actin network formation or function, the first option is to photoactivate actin monomers [87] or motors [88,89]. In this case, the activation is transient (defined by the illumination timing) and the size and shape of the activated area are controllable.

### Micropatterns

**Figure 1:**
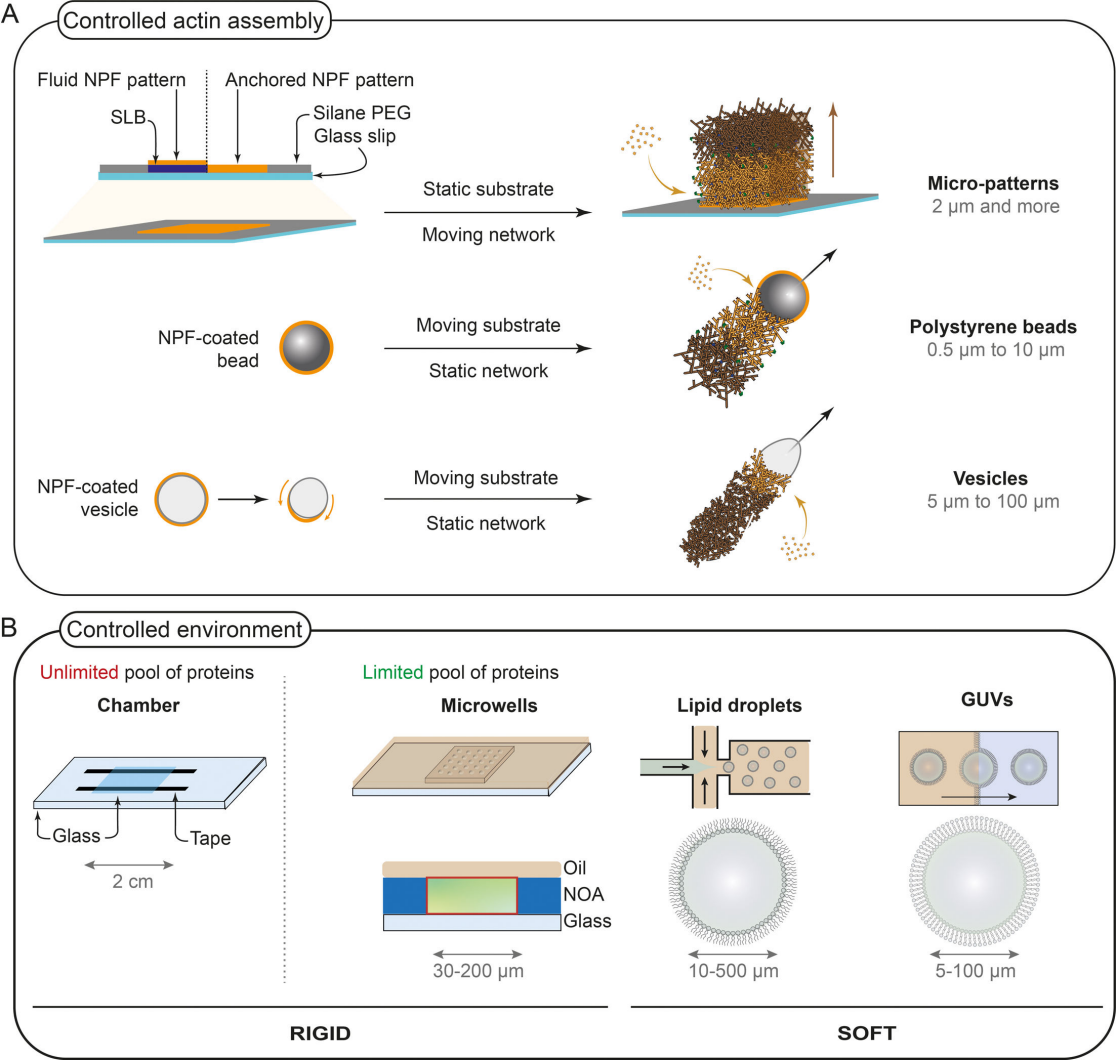
Toolbox for macroscopic reconstitutions. (**A**) Strategies for controlled actin polymerization in space and time. Top: micropatterns are obtained by burning a Silane PEG layer and then adding lipids and/or the NPF to localize the actin polymerization on the micropattern. Bottom: NPF can be coated on a bead (polystyrene or glass) or on a vesicle. The force generated by the actin network growth triggers the bead or vesicle movement. (**B**) Strategies for the design of confined environments with various sizes and properties. Abbreviations: NOA: Norland Optical Adhesive, GUVs: giant unilamellar vesicles. SLB: Supported Lipid Bilayer. NPF: Nucleation-Promoting Factor.

Micropatterning consists of burning a selected region on a passivated surface, in order to create “spots” of actin polymerization. Reymann *et al.* [90] developed this method in order to localize a nucleation-promoting factor (NPF) and generate branched networks of various shapes in controlled locations. The localization of the NPF triggers the generation of specific architectures in 2D [91,92] or in 3D [93,94]. More recently, the development of micropatterns covered with lipid bilayers allowed to mimic the activation of actin polymerization close to a membrane [95–97]. In the case of micropatterns, the activation is permanent and static while size and shape of the pattern remain controllable during the burning stage (Figure 1A).

### Beads and vesicles

The reconstitution of actin networks around beads or vesicles comes from the historical reconstitution of the sustained motility of the bacterium *Listeria monocytogenes* with purified proteins [98]. The reconstitution of actin structures around beads was first done in egg extracts [19,99–101] and then with purified proteins [102]. By coating the bead with an NPF and putting it in the appropriate mixture of proteins [102,103], a comet of branched actin filaments grows from the bead, with actin polymerization propelling the bead forward (Figure 1A). One of the main advantages of this assay is that the movement of the bead provides a reliable readout of actin dynamics. Additionally, the size, shape, and intensity of the network can be easily measured using fluorescence microscopy [84]. Activation is permanent as the NPF coating the bead is constitutively active, and bead size and shape can be modified and controlled [77,101]. The reconstitution of motility using NPF-coated vesicles in egg extracts has been used to probe the mechanical forces exerted by F-actin networks on membranes (Figure 1A [104,105]).

### Various compartments can be used for mechanical constraints and component limitations

Microwells (or microfabricated chambers, Figure 1B) have been used for a long time to create 3D confinement of microtubules [106]. They can be constructed using lithography methods, and the walls of the chambers can be easily functionalized with specific proteins or lipids [106,107]. Actin has been successfully encapsulated in microfabricated chambers [79,108].

Vesicles (or liposomes) are based on a lipid bilayer made of phospholipids (Figure 1B). They are currently the first choice of encapsulation for people working in the field of synthetic cell (which aims to understand how cells work by reproducing their molecular mechanisms [109,110]), and actin has also already been successfully encapsulated in liposomes [111]. However, even if various methods were developed to produce the vesicles, protein encapsulation is challenging and not always reproducible [83,112–114]. Another alternative is to use water-in-oil droplets (Figure 1B), which can be more easily created and filled with proteins. Phospholipids are usually dissolved in oil to stabilize the emulsion interface: they act as a surfactant, reducing the surface tension between the oil and water phases, and thus reducing droplet coalescence [115]. With this method, the outside medium being oil, exchanges with the outside medium are prohibited. The main advantage of droplets and vesicles is that they can be deformed [80] to modify their shape and see the influence on protein organization. Therefore, they are a potent tool, in the field of synthetic cells, to tackle the challenges of the reconstitution of motility and cell division, for example [116].

### Mimicking the proximity with a membrane: lipid bilayers

Many processes related to actin cytoskeleton function happen close to a membrane. Supported lipid bilayers (SLBs) are a powerful system to mimic the plasma membrane [117]. They consist of a glass coverslip functionalized with various lipids that are separated from the substrate by a thin layer of water to allow the lateral mobility of lipids. They can be easily imaged with TIRF microscopy and they have been combined with the other tools described above to create lipid micropatterns, for example [96].

### Combination of these approaches

Interestingly, all the above approaches can be used in combination. Indeed, SLBs have been adapted for the design of lipid micropatterns to study the effect of frictional forces on actomyosin contractility [96], NPF-coated beads or micropatterns can be introduced into microwells to study the effect of a limited amount of components [84,85], and giant unilamellar vesicles (GUVs) can be spread on micropatterns to see the influence of vesicle shape on actin orientation [118,119]. The use of various lipid compositions that spontaneously segregate into domains also makes it possible to target actin polymerization in a specific domain on GUVs [120].

## What kind of questions have been and can be addressed using macroscopic reconstitutions?

### Influence of actin architecture on various processes

Context. In cells, various architectures coexist and share the same pool of actin monomers and actin-binding proteins [6,7,121]. Understanding how the same pool of proteins can mediate various functions in the cells has been a key question in the actin cytoskeleton field for the last decade.

**Figure 2:**
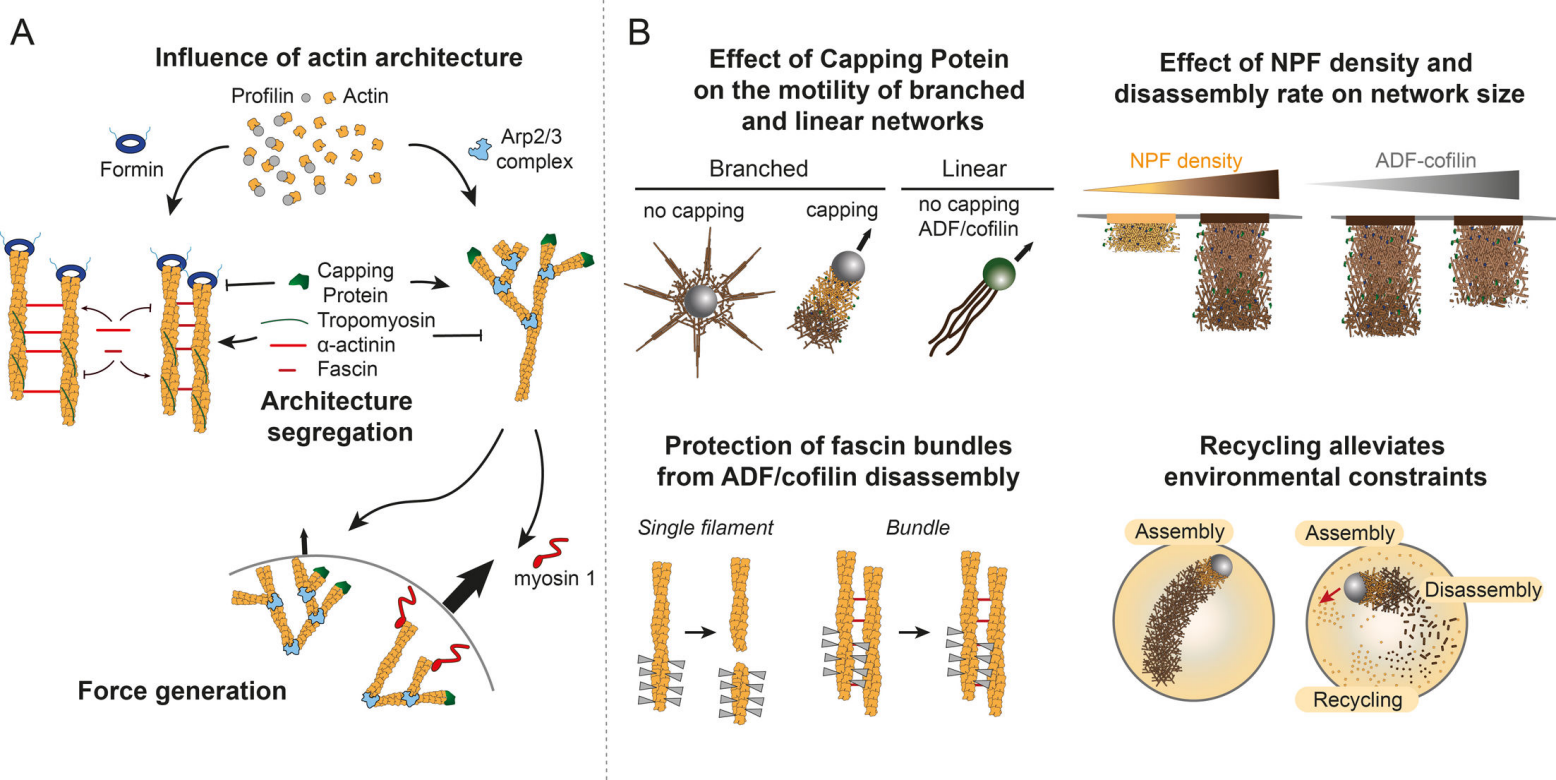
Mechanistic details obtained on the influence of actin architecture and turnover using macroscopic reconstructions. (**A**) Actin architecture (linear bundles or branched network) has been shown to influence protein binding such as capping protein or tropomyosin and alpha-actinin. (**B**) Actin turnover has been looked at from several angles. Motility of branched or linear networks was reconstituted from different components. Then, NPF density and disassembly rate were shown to influence the size of branched networks. Fascin-cross-linked bundles are protected from ADF/cofilin disassembly. Finally, recycling has been shown to be necessary for long-lived branched networks in a dynamic steady state.

Reconstitution of various architectures on micropatterns has made it possible to investigate the role of several factors on network contractility and how it leads to global shape changes. Indeed, architecture (branched or linear network [91,92]), connectivity (degree of filament cross-linking [92,122]), boundaries (zone in which myosin is active [88]), or friction between the actin network and its interacting substrate [96] are all factors that influence global actin network contraction. Large-scale reconstitution of various architectures on lipid bilayers gave insights into the impact of network architecture on the generation of stresses [123] and showed that contractility is highly cooperative and telescopic (i.e. that the speed of contraction is proportional to the size of the region where myosin activity has been activated) [89]. By reconstituting actomyosin networks in oil droplets, Sakamoto and Murrell [124] showed the impact of network architecture on energy consumption by myosin. In a similar fashion, reconstitutions on micropatterns allowed to show the selectivity of actin architecture on disassembly of the network [125] and the impact of network density on Actin Depolymerization Factor (ADF)/cofilin-mediated disassembly [126] (Figure 2B).

The binding and localization of proteins have also been addressed in the context of the competition between branched and linear networks thanks to the reconstitution of branched and linear networks on polystyrene beads [127,128]. In addition, reconstitution of branched and linear actin networks on beads has demonstrated that profilin—an abundant actin monomer-binding protein that inhibits spontaneous filament polymerization—restricts the growth of branched networks while promoting the growth of linear networks [129,130]. With the same system, Suarez *et al*. and Winkelman *et al*. [131,132] showed how protein competition and sorting depend on actin architecture (Figure 2A).

The reconstitution of branched networks on micron-sized beads has also provided valuable insights into the functional interaction between the various molecular players involved in these networks [133–139]. On beads’ surface, two different proteins can be localized, therefore allowing to decipher the effect of proximity between proteins (and therefore subsequent binding) on the formation of dendritic networks. For example, by co-localizing lamellipodin and VCA on a glass bead coated with a lipid bilayer, Hansen and Mullins [140] proposed that lamellipodin slows the assembly of branched networks. In another study, by functionalizing polystyrene beads simultaneously with an activator of the Arp2/3 complex and Myosin-I, Xu *et al.* [141] showed that myosin I can boost the force-generation capacity of a branched network (Figure 2A). All these examples show that reconstitution of actin networks on micropatterns or around micron-sized beads is a versatile system to address the effect of actin architecture on the binding and localization of actin-related proteins.

Perspectives. Taking into account, in the reconstitution, the association of actin-binding proteins on various actin isoforms and on actin with various post-translational modifications will certainly be of primary importance to understanding the formation of networks with various biochemical identities in the cell [142]. In addition, how the shape and function of actin networks evolved and diversified in the eukaryotic lineage is still poorly understood [1]. Understanding how different organisms have evolved different actin dynamics for their specific functions has begun to be addressed with structural and single-filament methods [143–145] but could now easily be tackled at the network scale. This will make it possible to assess the impact of these different dynamics on the functions and properties of different actin networks [1,146].

### Interaction between actin and microtubule networks

It is well known that the interaction of actin filaments with microtubules is critical for several cellular functions [147]. The set-up of biochemical conditions to reconstitute simultaneously dynamic actin networks and dynamic microtubules [148] was key for showing how actin network architecture regulates microtubule growth and dynamics [53,149,150], how actin filaments can guide microtubule organization or movement and act as a structural memory [151,152], and how actin architecture can mediate centrosome positioning [107] without the need for cross-linker between the two networks. Engineering of a protein that links growing microtubule ends to actin filaments (TipAct [51]), showed that growing microtubules can be captured and guided by stiff actin bundles, leading to global actin-microtubule alignment. Thus, the simultaneous reconstitution of actin and microtubule networks has shown that actin can impose its organization on microtubules, and vice versa.

Perspectives. The molecular mechanisms behind the effects of actin–microtubule interaction mentioned above are not yet well understood. The hypothesis of a frictional force between actin and microtubule fibers has been put forward to explain the impact on polymer dynamics when the two networks align [53,54] but still needs further validation. In addition, future works studying the positioning of the two networks in relation to each other and the potential impact on signaling [21] are exciting avenues to better understand the cross-talk between those two networks in the cell.

### Dynamic/turnover of structures

Context. The dynamic nature of actin architectures is of paramount importance when cells have to adapt to a changing environment [153–155]. In cells, the different coexisting architectures have various turnover rates, depending on their functions, with very fast turnover rates for lamellipodial or endocytic structures and slower turnover rates for stress fibers, for example [156]. However, understanding how these different turnover rates are established and maintained remains a challenge, and how the assembly and disassembly rates are coordinated to maintain a constant structure size through various feedbacks is still unclear. Therefore, the reconstitution of structures in a dynamic steady state is of primary importance to understand the basic mechanisms of actin turnover [156].

Mc Call *et al.* [157] reconstituted entangled actin filaments and showed the impact of nonequilibrium turnover on the dynamics and mechanics of the filaments. In particular, they demonstrated that the severing protein ADF/cofilin promotes fluidization of the network. On another hand, Guo *et al.* [158] showed that actin-binding proteins CAP (Cyclase-associated Protein) and Abp1 (Actin-binding protein 1) can drastically coalesce branched actin networks into bundles.

Studies reconstituting branched or linear actin networks on beads or micropatterns have provided valuable insights into the different steps of the turnover cycle. Reconstitution of branched networks around WASP-coated beads showed the minimal ingredients necessary for the formation of star-like networks, resembling the filopodia found in cells [100] (Figure 2B). The reconstitution of bacteria motility with purified proteins showed the dependence on the capping protein for the motility velocity [98]. Further work on branched networks reconstituted on beads demonstrated how capping protein increases the rate of motility by promoting more frequent filament nucleation [134]. Bead motility assays also allowed to point out the key role of ATP hydrolysis in branched actin network assembly and disassembly [84,159]. Interestingly, the formin-based motility does not require capping protein, which even causes arrest of formin-coated beads [160] (Figure 2B). Regarding the disassembly step, reconstitution of actin turnover on beads or on micropatterns has been instrumental in understanding how stochastic severing by ADF/cofilin facilitates the network turnover by releasing large parts of the actin bundles [38,103] and how the disassembly rate depends on the actin network density and architecture as well as on cofilin concentration [125,126]. By reconstituting linear bundles cross-linked by fascin, Chikireddy *et al.* [161] showed that fascin cross-linking protects the bundles from ADF/cofilin disassembly (Figure 2B). Moreover, Pollard *et al.* [162] identified that a mixture of seven purified proteins was sufficient to reconstitute cable formation with a polarized turnover. Finally, coating of beads with NPF for branched actin networks as well as formins showed a synergic activity of the two proteins and a formin-mediated protection from debranching by ADF/cofilin [163]. Until recently, the recycling step has been overlooked in macroscopic reconstitutions. However, recent studies combining bead motility assay and confinement in microwells demonstrated how recycling is necessary to ensure long-lived turnover of actin networks, and how this turnover allows several competitive structures to coexist in the same environment [84,85].

Perspectives. The different steps of actin turnover (assembly, disassembly, and recycling) are now well understood at the molecular level. Even if some studies started to address their coordination with reconstituted systems, how these steps are balanced and, more precisely, what types of feedback exist between them is still unclear and requires more exploration. The hypothesis that the size of the available actin monomer pool serves as feedback for the assembly rate has been proposed by different studies in cells [129,164] and in reconstituted systems [84] but still needs further validation. Furthermore, understanding how architectures with different sizes and turnover rates share the same limiting pool of proteins remains a key unanswered question. Theoretical studies have proposed possible mechanisms behind dynamic scaling [165–169] and the reconstituted systems at the mesoscale mentioned above have the power to test the validity of those mechanisms. More precisely, with the use of confined environments, it is possible to finely tune the number of proteins, as well as their concentration or density, and the physical properties of the medium, and therefore to study the impact of these factors on the size and dynamic of different actin architectures.

### Mechanical properties of actin networks

Context. Cells are intrinsically subjected to several mechanical constraints, and they also produce forces during the processes of morphogenesis, migration, endocytosis, organelle transport, or cell division [7]. Historical reconstitutions of entangled and branched networks and their analysis with passive or active micro-rheology tools have been key to understanding the viscoelastic properties of actin networks [170–172]. Addition of myosin and cross-linkers to these networks allowed to show that contractility occurs at optimal concentrations of motors and cross-linkers [92,173–175].

**Figure 3:**
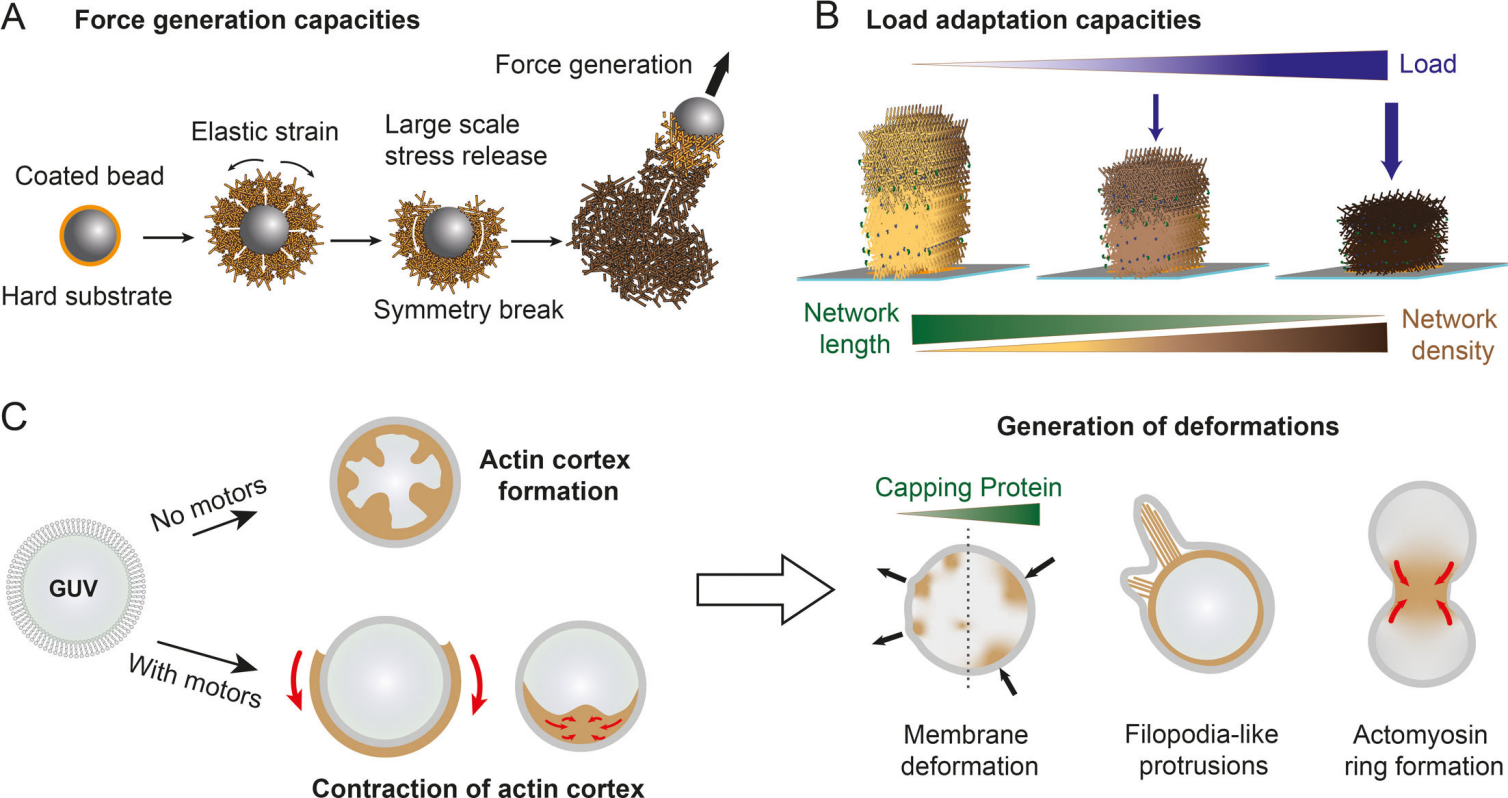
Mechanistic details gained on mechanical properties of actin networks and on their interaction with the membrane using macroscopic reconstitutions. (**A**) When coated with an NPF and placed in a mix of purified proteins, an actin network grows around the beads and then accumulates elastic strain. The release of this stress triggers a symmetry breaking and therefore the bead movement. (**B**) Networks grown from micropatterns in three dimensions were shown to adapt their density, depending on the applied load. (**C**) Actin networks were polymerized inside and outside of GUVs and were shown to break their symmetry in the presence of molecular motors. Depending on the protein present in the experiment, membrane deformation, filopodia-like protrusions, or actomyosin rings can be observed.

Then, the reconstitution of actin networks around beads or vesicles has been key to obtaining a better understanding of the forces generated by a growing actin network. Indeed, by first using beads coated with ActA and introduced in HeLa cell extracts, Noireaux *et al.* [176] estimated that forces exerted by an actin gel are about 10 pN. Subsequent studies with purified proteins showed the impact of physical properties on the growth of branched networks [77,102] and the tight coupling between biochemical and physical properties of the networks [177,178]. Moreover, this type of assay allowed to show that the symmetry breaking required to move the bead is based on the release of elastic energy, analogous to the fracture of polymer gels [179,180] (Figure 3A) and that the elastic gel exerts a friction force on the bead [181,182]. Reconstitution of motility on deformable objects (GUVs) coated with an activator of the Arp2/3 complex showed that the deformability of the object and the mobility of the activator at the lipid surface affect dynamic and structural parameters of the actin networks [183,184]. Altogether, these assays provide a better understanding of the mechanisms underlying motility initiation [134,185].

Reconstitution of branched actin networks growing from micropatterns in 3D, combined with an AFM tip to apply force on the growing network, allowed showing in a very elegant manner that growth of a branched network against a load leads to a denser network and to an adaptation of the architecture [70,71](Figure 3B), similarly to what has been described in cellular systems [153,186]. In addition, the growth of branched networks in 2D from similar micropatterns with different densities of NPF led to the generation of heterogeneous networks; this network heterogeneity was shown to impose a direction in motility [187].

The generation of forces relies a lot on the adhesive properties of the cells. This aspect has been studied by using micropatterns functionalized with the talin protein. These studies revealed that actomyosin-dependent dynamics of the talin–vinculin complex and activation of vinculin by stretched talin induce a positive feedback that reinforces the actin–talin–vinculin association, and that actomyosin force triggers protein binding on the mechanosensitive protein talin [188,189]. Study of vinculin’s interaction with branched networks grown from micropatterns showed that vinculin can bundle dendritic actin networks at nascent adhesions [190].

Perspectives. Altogether, the experiments on beads and on micropatterns shed some light on the mechanical properties of branched actin networks, a key feature for motility, endocytosis, and more. In the next years, the reconstitution of motility from purified proteins will certainly help to understand the minimal elements necessary for cell movement and the mechanisms associated with different types of migration [191]. In this context, in order to understand the molecular mechanisms and minimal requirements for cell motility, the reconstitution of adhesion will be of primary importance. First steps toward this adhesion reconstitution has been done by binding talin at the surface of GUVs and by looking at the effect of the presence of talin, as well as kindlin and actomyosin, on the ability of the system to cluster integrins [192].

### Actin interaction with membrane (2D and 3D)

Context. Interaction between actin and the plasma membrane plays a key role in many biological processes (cell division, cell migration, endocytosis, cell spreading, membrane trafficking, long-range coordination of cell polarity, etc.) [193–198]. Actin is present below the membrane (forming the cortex) and the plasma membrane is a big spot of actin polymerization regulation (via the Pip2, Rho pathways, etc.). For a cell to move, actin assembly must generate a force sufficient to move the plasma membrane. There is a reciprocal interaction between actin and the cell membrane: indeed, actin polymerization can increase membrane tension but actin polymerization can also be triggered at the curved regions (via the recruitment of BAR proteins, for example) [197].

Many studies have reconstituted actin networks close to lipid membranes [117]. By using SLBs in two dimensions, Murrell and Gardel, and Vogel *et al*. [199,200] showed that the extent of actin adhesion to a membrane can regulate the coupling between network contraction and F-actin severing. In more recent studies, variation of lipid composition was shown to have an impact on the connectivity to the membrane [201] and VASP (Vasodilator-stimulated phosphoprotein) binding on lipids was shown to facilitate the formation of large bundles [202]. By adding myosin II to an actin cortex polymerized on a lipid bilayer, Sonal *et al.* [203] observed a dynamic reorganization of the actin network. In addition, it was shown that linkage between branched actin network and lipids can modulate the friction and orient the axis of contraction [96]. On the other hand, it was also shown that actin polymerization can reorganize lipids (and promote phase separation) [204–206]. Indeed, the use of SLB allows to see how large-scale reorganization of proteins can have an impact on actin polymerization. For example, Banjade *et al.* [207] showed that attachment of nephrin to a lipid bilayer promotes multivalent interactions and then phase separation of the protein with its cytoplasmic partners Nck and N-WASP. These clusters can then assemble branched actin network, therefore giving hints about the regulation of local actin assembly at membranes.

In three dimensions, the interactions between actin and membranes have mainly been studied with GUVs [208] but water-in-oil emulsions stabilized with lipids are also a good option to study actin–membrane interactions. Many studies focused on the generation of cortex-like structures outside or inside the vesicles (Figure 3C). The study of Pontani *et al.* [209] was the first to reconstitute an actin cortex inside a liposome. Next, Carvalho *et al.* and Loiseau *et al.* [210,211] added myosin to the cortex-like structures to generate cortical tension, the level of which is determined by the amount of motors, the connectivity of the network, and its attachment to the membrane. They observed remodeling of the actin cortex as well as changes in membrane shape (Figure 3C). Subsequent studies looked at the effect of actin-binding proteins like cross-linkers [212–214] or capping protein [215] on vesicle shape. Altogether, these studies demonstrated the importance of actin network connectivity and membrane attachment to drive large-scale shape changes (Figure 3C). Those shape changes of the vesicles have also been widely studied in the context of protrusion generation. For example, Liu *et al.* [216] showed that branched actin networks can deform a liposome membrane in “filopodia-like” bundled actin protrusions. Then, Simon *et al.* and Allard *et al*. [217,218] demonstrated that reconstitution of branched actin network (with capping protein) is sufficient to drive inward and outward membrane deformation. Moreover, heterogeneities in the actin network can favor membrane protrusions [219]. Actin networks can sense membrane curvature [83], and actin rings formed inside water-in-oil droplets spontaneously positioned at the equator of the droplet [220]. Recently, a few studies showed that actomyosin rings, targeted at the equatorial plane of vesicles, could deform the vesicles [221,222]. Moreover, addition of a membrane curvature sensor can recruit the actin polymerase VASP to assemble actin filaments locally on membranes and then generate protrusions that are similar to filopodia [223].Therefore, reconstitution of assembly-based actin network inside deformable vesicles revealed a large range of deformations, which can be compared with those observed in cells. In addition, different methods give quantitative measures of the magnitude of those forces, which is necessary for a mechanistic understanding of the actin-based processes associated with the membrane.

Interaction of actin with membrane also happens in cargo transport where most of the cargos are vesicles. Interaction of actin filaments with motors transporting cargos is important for polarized transport of material in the cell [224]. By reconstituting liposomes transported by myosin Va in a 3D actin network, Lombardo *et al*. [225] demonstrated the impact of actin architecture, and therefore of actin filament polarity, on the transport of liposomes. Moreover, reconstitution of composite actin–microtubules networks with myosin1C and kinesin1 motors showed the influence of the motors on the vesicles deformation and therefore on the tubule formation [226].

Perspectives. As discussed in [227], there is still the need to precisely decipher the mechanisms of actin assembly at the membrane, and more precisely the mechanisms of the interaction between actin-membrane linkers and actin-associated proteins for the regulation of polymerization. In addition, understanding how actin-membrane linkers propagate membrane tension will give better insights for all the processes involving actin–membrane interaction. Moreover, in the field of synthetic cell development, reconstitution of dynamic actin networks inside vesicles is still a challenge. Reconstituting networks able to generate sufficient forces to divide the vesicle is an even bigger but exciting challenge.

## Future perspectives

Thanks to advances in biochemistry, the study of single filaments, and electron microscopy, many of the molecular players involved in actin structures are now well characterized and their functions better understood. With the tools described above, it is now conceivable to reconstitute any actin architecture under physicochemical conditions that mimic those found in cells, using a limited number of components. This approach could make it possible to identify the minimal set of ingredients required for key cellular processes such as motility, endocytosis, and division. However, a major limitation is the number of proteins that can be introduced simultaneously into a closed environment. Beyond the technical challenge of precise pipetting, the incorporation of multiple proteins leads to an exponential increase in the number of possible concentration combinations, which can exceed practical experimental capabilities. To meet this challenge, predictive mathematical models could prove invaluable. These models would make it possible to identify the key parameters to be varied, thus reducing the space of parameters to be experimentally tested. They would also make it easier to interpret the data, which becomes increasingly complex as the number of proteins and reactions in the assay increases. Alternatively, to test multiple combinations of parameters and protein concentrations, automated systems could be developed. Automation would allow precise pipetting and enable parallel experimentation, significantly increasing throughput and efficiency.

As mentioned above, the incorporation of physical parameters in the reconstitutions will certainly be of primary importance. Indeed, parameters like osmolarity, viscosity heterogeneity in the cytoplasm, or pH gradients were shown to be crucial for cytoskeleton-based processes in cellular studies [74,228–230]. Moreover, a cell is not a homogeneous medium, so incorporating heterogeneity into reconstituted systems will also be of paramount importance. This can be achieved by confining some reactions to certain compartments (with or without membrane) [231,232] or by incorporating spatio-temporally controlled signaling [21,233–236]. On a longer and more technological timescale, reconstitution of actin at the macroscopic level could find applications in electronics [93] or in living materials fields [237].

The combination of studies reconstituting networks at the macroscopic level, with work studying actin at the single-molecule or single-filament level, and with research studying actin at the cellular or organismal level, provides a multi-scale approach and powerful tools for linking the different scales of actin complexity. The use of these complementary approaches will certainly enable us to fully answer the questions mentioned in the introduction in the years to come.

## Abbreviations

GUV: giant unilamellar vesicles
NOA: Norland Optical Adhesive
NPF: nucleation-promoting factor
SLB: supported lipid bilayer
